# Detection of Real-Time Changes in Direction of COVID-19 Transmission Using National- and State-Level Epidemic Trends Based on *R_t_* Estimates — United States Overall and New Mexico, April–October 2024

**DOI:** 10.15585/mmwr.mm7346a3

**Published:** 2024-11-21

**Authors:** Danielle M. Richard, Zachary Susswein, Sarah Connolly, Adán Myers y Gutiérrez, Roselyn Thalathara, Kelly Carey, Emily H. Koumans, Diba Khan, Nina B. Masters, Nathan McIntosh, Patrick Corbett, Isaac Ghinai, Rebecca Kahn, Adrienne Keen, Juliet Pulliam, Daniel Sosin, Katelyn Gostic

**Affiliations:** ^1^Inform Division, Center for Forecasting and Outbreak Analytics, CDC; ^2^Predict Division, Center for Forecasting and Outbreak Analytics, CDC^: 3^New Mexico Department of Health; ^4^Detect and Monitor Division, Office of Public Health Data, Surveillance and Technology, CDC; ^5^Coronavirus and Other Respiratory Diseases Division, National Center for Immunization and Respiratory Diseases, CDC; ^6^Division of Viral Diseases, National Center for Immunization and Respiratory Diseases, CDC; ^7^Booz Allen Hamilton, Atlanta, Georgia.

**Summary**

**What is already known about this topic?**

Surveillance data are often subject to delays, which can affect the ability of public health decision-makers to conduct accurate real-time assessments.

**What is added by this report?**

During summer 2024, trend categories that incorporated nowcasting provided an early indication that COVID-19 community transmission was increasing and later, provided confirmation that community transmission was decreasing.

**What are the implications for public health practice?**

Epidemic trend categories that incorporate nowcasting can provide early indication of changing trends in community transmission and can help public health decision-makers quickly determine whether transmission of infection is growing or declining at a given time and prepare for public health action.

## Abstract

Public health practitioners rely on timely surveillance data for planning and decision-making; however, surveillance data are often subject to delays. Epidemic trend categories, based on time-varying effective reproductive number (*R_t_*) estimates that use nowcasting methods, can mitigate reporting lags in surveillance data and detect changes in community transmission before reporting is completed. CDC analyzed the performance of epidemic trend categories for COVID-19 during summer 2024 in the United States and at the state level in New Mexico. COVID-19 epidemic trend categories were estimated and released in real time based on preliminary data, then retrospectively compared with final emergency department (ED) visit data to determine their ability to detect or confirm real-time changes in subsequent ED visits. Across the United States and in New Mexico, epidemic trend categories were an early indicator of increases in COVID-19 community transmission, signifying increases in COVID-19 community transmission in May, and a confirmatory indicator that decreasing COVID-19 ED visits reflected actual decreases in COVID-19 community transmission in September, rather than incomplete reporting. Public health decision-makers can use epidemic trend categories, in combination with other surveillance indicators, to understand whether COVID-19 community transmission and subsequent ED visits are increasing, decreasing, or not changing; this information can guide communications decisions.

## Introduction

Epidemiologists, public health practitioners, and the public rely on surveillance data to guide decisions and plan public health interventions. Data timeliness is a critical factor in making accurate real-time assessments; however, surveillance data are often subject to delays, including biologic delays, from the time a person is infected until an observable event occurs (e.g., positive test result, emergency department [ED] visit, or hospitalization), and reporting delays, from the time the observable event occurs until the event is reported to local health jurisdictions. Such delays make it difficult to determine whether infections are increasing, not changing, or decreasing until after all data have been fully reported, which might be days to weeks after the events of interest have taken place. Because the number of infections or events at a given point in time is unknown, certain surveillance metrics, such as ED visits, can provide information about trends in community transmission.

In May 2024, the Center for Forecasting and Outbreak Analytics, in collaboration with the National Center for Immunization and Respiratory Diseases and the National Syndromic Surveillance Program (NSSP), began publishing weekly epidemic trend categories for COVID-19, based on time-varying effective reproductive number (*R_t_*) estimates and nowcasted ED visit data online.^§^ In advance of the 2024–25 respiratory virus season, CDC analyzed the performance of weekly national epidemic trend categories and collaborated with the New Mexico Department of Health to determine state-level epidemic trend categories for COVID-19 compared with raw ED visit data during April 19–October 18, 2024.

## Methods

### Data Source and Nowcasting

Epidemic trend categories are determined based on *R_t_* (i.e., the average number of new infections caused by each infectious person at a particular time), calculated from nowcasted ED visit data from NSSP^¶^ (*1*,*2*). Nowcasting is a mathematical approach that addresses delays in surveillance data and associated uncertainty and can be used to estimate the final number of ED visits based on currently available, partially reported data and historic patterns of reporting delays (*3*–*5*). To adjust for reporting delays, a statistical nowcasting correction was applied to daily ED visit counts from the previous 30 days.**

### Classification of Transmission Direction Using *R_t_*

*R_t_* indicates whether disease transmission is increasing or decreasing. If *R_t_* >1, the number of new infections is increasing; if *R_t_* = 1, the number is not changing; and if *R_t_* <1, the number of new infections is declining. *R_t_* was estimated from the nowcast of COVID-19 ED visits^††^ using the statistical package EpiNow2 (version 1.4.0; EpiForecasts) in R (version 4.3.1; R Foundation) with parameters as previously described (*6*,*7*). EpiNow2 estimates *R_t_* and the probability that *R_t_* >1, *R_t_* = 1, or *R_t_* <1. The epidemic trend category was determined based on the probability that *R_t_* >1 (i.e., the probability that each infectious person spreads the virus to more than one other person). The epidemic trend category was classified as growing (probability >90%), likely growing (>75% to ≤90%), not changing (>25% to ≤75%), likely declining (≥10% to ≤25%), or declining (<10%).^§§^ Epidemic trend categories were published online each Friday based on daily COVID-19 ED visit counts through the preceding Tuesday, for the United States overall and for all 50 states and the District of Columbia^¶¶^ (*2*).

### Comparison of Weekly Epidemic Trends with ED Visits

Weekly epidemic trend categories were compared with reported weekly percentages of COVID-19–related ED visits in the United States*** and with reported number of daily COVID-19–related ED visits in New Mexico^†††^ at four points during summer 2024: 1) before an increase in ED visits for COVID-19 (May 10), 2) during the increase (July 5), 3) near the peak (September 6), and 4) after the peak (October 18). This activity was reviewed by CDC, deemed not research, and was conducted consistent with applicable federal law and CDC policy.^§§§^

## Results

### U.S. COVID-19 ED Visits and Epidemic Trends

**FIGURE 1:**
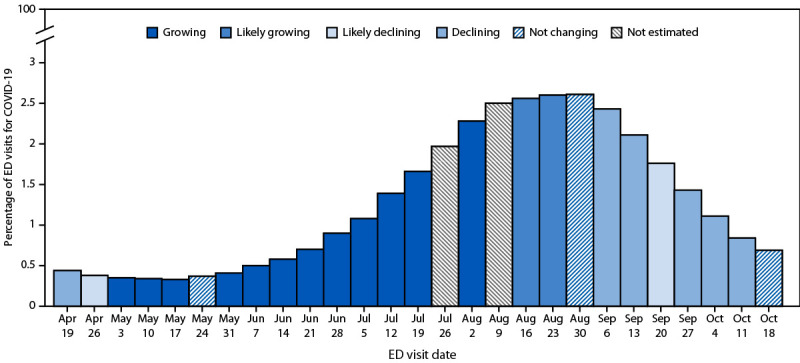
Percentage of emergency department visits for COVID-19, by date and weekly epidemic trend category* — United States, April–October 2024 **Abbreviation:** ED = emergency department. * The percentage of ED visits for COVID-19 are publicly available data, displayed by the date the data become available (Friday), using data from the previous surveillance week (ending the previous Saturday). Publicly available data for the national percentage of COVID-19–related ED visits might differ from data used to estimate *R_t_*, because of differing inclusion and exclusion criteria (e.g., a static subset of all facilities reporting to NSSP is used for *R_t_* estimation). The *R_t_*–based epidemic trend categories are presented by date of *R_t_*-estimate publication (Friday), which is calculated from a nowcast of ED visit data reported through the previous Tuesday. Therefore, although both percentage of ED visits and *R_t_* estimates are published weekly on Friday, the *R_t_* estimate calculation reflects 3 additional days of recent ED visit data.

In summer 2024, the United States experienced an increase in COVID-19 diagnoses. For the week of May 10, 2024, epidemic trend categories for the United States and for 11 states on the West Coast and in the Northeast began to indicate a growing or likely growing trend, although the percentage of reported COVID-19–related ED visits in the United States at this time was low (0.3%), ranging from 0.3% to 1% in these 11 states (Figure 1) (Supplementary Figure, https://stacks.cdc.gov/view/cdc/169819) (Table). For the week of July 5, 2024, epidemic trend categories were growing for the United States overall and growing or likely growing in 39 states. During the same week, the reported percentage of COVID-19–related ED visits was 1.0% and ranged from 0.4% to 2.4% in these 39 states.

**TABLE:** Percentage of COVID-19–related emergency department visits* and epidemic trend categories**^†^****^,^****^§^** at four points during the summer 2024 COVID-19 wave, by U.S. jurisdiction — National Syndromic Surveillance Program, United States, May 10, July 5, September 6, and October 18, 2024

Jurisdiction	May 10, 2024		Jul 5, 2024		Sep 6, 2024		Oct 18, 2024	
	COVID-19 ED visits, %	Epidemic trend	COVID-19 ED visits, %	Epidemic trend	COVID-19 ED visits, %	Epidemic trend	COVID-19 ED visits, %	Epidemic trend
**United States**	**0.3**	**Growing**	**1.0**	**Growing**	**2.3**	**Declining**	**0.7**	**Not changing**
Alabama	0.3	Not changing	0.8	Growing	3.3	Declining	0.6	Not changing
Alaska	0.3	Not changing	1.5	Not changing	1.8	Not changing	0.5	Declining
Arizona	0.5	Likely growing	1.7	Not changing	1.7	Not changing	1.0	Likely declining
Arkansas	0.3	Not changing	0.5	Growing	3.0	Not changing	0.5	Declining
California	0.3	Likely growing	1.5	Growing	2.1	Likely declining	0.5	Declining
Colorado	0.4	Likely growing	1.0	Growing	1.8	Not changing	1.0	Declining
Connecticut	0.3	Not changing	0.5	Likely growing	1.4	Declining	0.6	Not changing
Delaware	0.5	Not changing	0.8	Likely growing	2.4	Not changing	0.6	Declining
District of Columbia	0.4	Not changing	0.9	Growing	1.9	Not changing	0.3	Declining
Florida	0.4	Not changing	2.4	Growing	2.4	Declining	0.5	Likely declining
Georgia	0.2	Not changing	0.6	Growing	2.1	Declining	0.3	Likely declining
Hawaii	1.0	Growing	4.3	Not changing	2.0	Not changing	0.6	Declining
Idaho	0.3	Not changing	0.9	Growing	2.4	Growing	0.9	Declining
Illinois	0.3	Not changing	0.8	Growing	2.5	Declining	0.6	Declining
Indiana	0.2	Likely declining	0.6	Growing	2.8	Declining	0.6	Not changing
Iowa	0.3	Not changing	0.4	Likely growing	2.3	Not changing	0.8	Likely declining
Kansas	0.2	Declining	0.4	Growing	1.9	Not changing	0.7	Not changing
Kentucky	NA	Not estimated	0.6	Growing	3.6	Not changing	0.8	Not changing
Louisiana	0.2	Not changing	1.2	Growing	2.6	Declining	0.5	Likely declining
Maine	0.5	Likely growing	0.6	Likely growing	1.9	Likely declining	1.4	Declining
Maryland	0.4	Not changing	0.9	Growing	2.5	Not changing	0.6	Not changing
Massachusetts	0.4	Not changing	0.9	Growing	1.9	Not changing	0.8	Declining
Michigan	0.4	Declining	0.5	Likely growing	2.2	Growing	0.9	Likely declining
Minnesota	0.3	Not changing	0.9	Growing	2.0	Not changing	1.1	Declining
Mississippi	0.3	Declining	0.7	Growing	2.5	Likely declining	0.5	Declining
Missouri	NA	Not estimated	NA	Not estimated	NA	Not estimated	NA	Not estimated
Montana	0.4	Not changing	0.9	Growing	1.7	Likely growing	1.4	Declining
Nebraska	0.1	Likely declining	0.5	Growing	1.5	Not changing	0.5	Declining
Nevada	0.3	Likely growing	1.1	Likely growing	1.7	Not changing	0.7	Declining
New Hampshire	0.3	Not changing	0.6	Growing	2.1	Not changing	1.5	Likely declining
New Jersey	0.3	Likely growing	0.8	Not changing	1.5	Not changing	0.6	Declining
New Mexico	0.5	Growing	1.5	Not changing	2.5	Declining	1.2	Not changing
New York	0.3	Growing	0.9	Not changing	1.4	Likely declining	0.6	Not changing
North Carolina	0.3	Declining	0.8	Growing	3.4	Not changing	0.7	Likely declining
North Dakota	0.3	Not changing	0.6	Likely growing	1.4	Not changing	0.8	Likely declining
Ohio	0.3	Likely declining	0.5	Growing	2.6	Not changing	0.7	Not changing
Oklahoma	0.3	Not changing	0.5	Likely growing	2.5	Not changing	0.7	Declining
Oregon	0.4	Growing	1.6	Not changing	2.3	Not changing	1.3	Declining
Pennsylvania	0.2	Not changing	0.5	Not changing	1.9	Not changing	0.7	Not changing
Rhode Island	0.2	Not changing	0.4	Likely growing	1.3	Not changing	0.5	Declining
South Carolina	0.2	Not changing	0.8	Growing	3.4	Likely declining	0.5	Declining
South Dakota	NA	Not estimated	0.8	Growing	1.7	Growing	0.8	Likely declining
Tennessee	0.3	Not changing	0.6	Growing	2.6	Declining	0.5	Likely declining
Texas	0.3	Not changing	1.3	Growing	2.7	Declining	0.4	Declining
Utah	0.3	Not changing	0.9	Likely growing	2.1	Likely growing	0.9	Declining
Vermont	0.4	Not changing	0.7	Likely growing	2.3	Not changing	1.4	Declining
Virginia	0.3	Likely declining	0.9	Growing	3.3	Likely declining	0.7	Likely declining
Washington	0.5	Not changing	1.7	Not changing	2.2	Growing	1.2	Declining
West Virginia	0.4	Not changing	0.5	Not changing	3.9	Growing	1.2	Not changing
Wisconsin	0.4	Likely growing	0.7	Growing	NA	Not estimated	NA	Not estimated
Wyoming	NA	Not estimated	NA	Not estimated	NA	Not estimated	NA	Not estimated

**Abbreviations:** ED = emergency department; NA = not available; NSSP = National Syndromic Surveillance Program; *R_t_* = time-varying effective reproductive number.

* The percentage of all ED visits that were related to COVID-19 illness available at the time and used to estimate *R_t_*. Data for the percentage of COVID-19–related ED visits might differ from publicly available data, because of differing inclusion and exclusion criteria (e.g., a static subset of all facilities reporting to NSSP is used for *R_t_* estimation).

^†^ The *R_t_*-based epidemic trend categories are displayed by date of *R_t_* estimate publication (Friday). The percentage of COVID-19–related ED visits in a given week are displayed by *R_t_* report date (Friday), using data from the previous surveillance week (ending the previous Saturday).

^§^ The epidemic trend category was determined based on the probability that *R_t_* >1 (i.e., the probability that each infectious person spreads the virus to one or more other persons). The epidemic trend category was classified as growing (*R_t_* >90%), likely growing (*R_t_* >75% to ≤90%), not changing (*R_t_* >25% to ≤75%), likely declining (*R_t_* ≥10% to ≤25%), or declining (*R_t_* <10%).

After the summer wave peaked in late August (i.e., COVID-19 accounted for 2.6% of ED visits, reported on August 30), epidemic trend categories indicated that COVID-19 community transmission was decreasing across the country. For the week of September 6, 2024, trends in the United States overall and in 16 states were declining or likely declining. The nationally reported percentage of COVID-19–related ED visits was 2.3%, ranging from 1.4% to 3.4% in these 16 states.

### New Mexico COVID-19 ED Visits and Epidemic Trends

**FIGURE 2:**
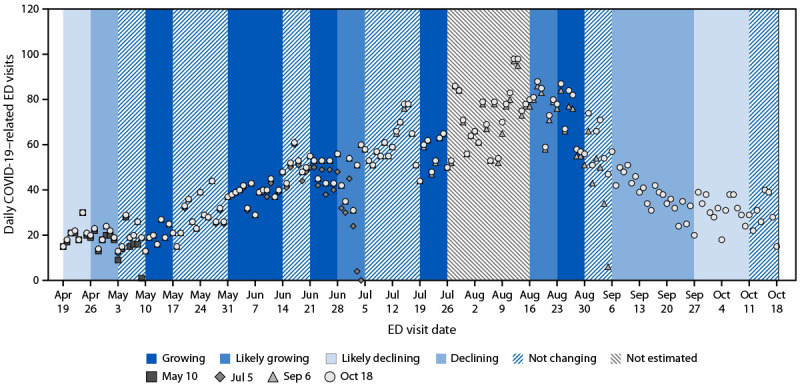
Number of COVID-19–related emergency department visits and epidemic trend categories — New Mexico, April–October 2024 **Abbreviation:** ED = emergency department.

In collaboration with the New Mexico Department of Health, state-level epidemic trend categories were assessed. The New Mexico epidemic trend categories provided an early signal of the summer wave. On May 10, 2024, the epidemic trend was growing; however, data on number of COVID-19–related ED visits available at that time did not yet demonstrate this increase. As of May 10, 99 COVID-19–related ED visits had been reported for the preceding 7 days, substantially fewer than the 130 such visits reported during the 7 days preceding May 3. However, the reports available on May 10 for the preceding 7 days included only 70% of the total 141 ED visits (as of October 18, 2024) (Figure 2).

In New Mexico, epidemic trend categories also confirmed that the summer wave had peaked and helped to distinguish actual decreases in transmission from apparent decreases resulting from incomplete reporting. From the week of May 3 to the week of August 30, the epidemic trend category was estimated to be either not changing or growing. The epidemic trend category was estimated to be declining for the first time since May 2024 during the week of September 6, 2024. ED visit data reported 304 COVID-19–related ED visits during the week of September 6 (7 days), compared with 489 such visits during the previous week.

## Discussion

During summer 2024, epidemic trend categories using nowcasted ED visit data served as early indicators of increasing COVID-19 ED visits and confirmatory indicators that COVID-19–related ED visits were either not changing or decreasing. In May, at the national level, epidemic trend categories accurately foreshadowed that infections and subsequent COVID-19–related ED visits would grow, before increases in ED COVID-19 visits were evident from surveillance data. In New Mexico, epidemic trend categories indicated increased community transmission in advance of complete reporting. Epidemic trend categories did not indicate early that COVID-19 ED visits would decrease or were decreasing in New Mexico. This finding might be related to when *R_t_* was calculated, from which the trend categories were derived, relative to when the decreases in COVID-19–related ED visits began that week. However, epidemic trend categories did confirm that decreases in reported ED visits reflected actual reductions in COVID-19 ED visits and did not represent delayed reporting.

Using a statistical nowcasting approach that provided an *R_t_* estimate and allowed for categorization of epidemic trends from incomplete data mitigated reporting delays inherent in surveillance data. When combined with other surveillance metrics, particularly those reflecting disease incidence, epidemic trend categories can provide useful information for public health preparedness and response. State-level trends provide local situational awareness and can be used by neighboring states to monitor regional trends. Trend categories can also help prepare health care providers for potential surges and enable public health practitioners to adjust prevention messaging to the public.

### Limitations

The findings in this report are subject to at least three limitations. First, although epidemic trend categories can indicate whether transmission is increasing or decreasing, they do not provide information about the total number of infections. Epidemic trend categories should, therefore, only be considered alongside other surveillance metrics that record disease incidence, such as ED visits, hospitalizations, and deaths. Second, CDC epidemic trend categories are currently published at the national and state levels and do not account for differences in community transmission at lower jurisdictional levels. Finally, ED visit data used for this analysis were subject to sporadic temporary data submission disruptions; these pauses increase reporting delays until the issues are resolved. CDC is exploring novel analytic approaches, such as combining multiple data sources, to reduce disruption in modeling when a single data source is temporarily interrupted.

### Implications for Public Health Practice

During summer 2024, epidemic trend categories, based on *R_t_* calculated from nowcasted ED visit data available through NSSP, identified growing trends in COVID-19 ED visits before increases were observable in raw surveillance data. Epidemic trend categories might reveal whether the number of infections is growing, not changing, or declining; however, they do not reflect the total number of infections and should be interpreted alongside other surveillance metrics. Epidemic trend categories provided early indication of changing trends in community transmission and are useful to prepare for public health action.

## Footnotes

* These authors contributed equally to this report.

† These senior authors contributed equally to this report.

§ The Center for Forecasting and Outbreak Analytics, in collaboration with the National Center for Immunization and Respiratory Diseases, began publishing weekly epidemic trend estimates using hospitalization data in November 2023. In spring 2024, because of changes in reporting requirements for hospitalizations, the data source for epidemic trend estimates transitioned to ED visit data from NSSP. In April 2024, epidemic trend estimates were produced internally and were published publicly beginning in May 2024.

¶ ED visits are reported to NSSP from approximately 80% of EDs in the United States and represent a stable metric from week to week. The proportion of persons with COVID-19 seeking treatment at an ED was assumed to be relatively constant during this evaluation.

** The statistical nowcasting correction is based on a jurisdiction-specific distribution, estimated from the average of observed reporting delays in the recent past, to adjust for incomplete reporting on recent dates.

†† COVID-19–related ED visits are based on the following *International Classification of Diseases, Tenth Revision, Clinical Modification* discharge diagnosis codes: J12.82 (pneumonia due to coronavirus disease 2019) and U07.1 (COVID-19 identified in an asymptomatic person) and the following Systematized Nomenclature of Medical Clinical Terms codes: 840539006 (disease caused by SARS-CoV-2), 840544004 (suspected disease caused by SARS-CoV-2), and 840533007 (SARS-CoV-2 [organism]).

§§ *R*_t_ was categorized as “not estimated” if fewer than 10 COVID-19–related ED visits were reported in each of the previous 2 weeks, if anomalies were detected in reported values, or if the model did not pass checks for reliability.

¶¶ Epidemic trend categories for many jurisdictions, including New Mexico, and for the United States, were not released for some or all weeks during July 26–August 9 because of a nationwide technological outage that temporarily interrupted data reporting in some jurisdictions.

*** Weekly percentage of COVID-19–related ED visits are publicly available data published weekly on Fridays. ED visit count data are not publicly available; however, count data are accessible to public health authorities in their respective jurisdiction. *R_t_* estimates are based on nowcasts of ED visit count data. National weekly percentages of ED visits are used as a comparison because these data are publicly available.

††† New Mexico has granted permission to publish its state-level ED visit count data for this assessment.

§§§ 45 C.F.R. part 46, 21 C.F.R. part 56; 42 U.S.C. Sect. 241(d); 5 U.S.C. Sect. 552a; 44 U.S.C. Sect. 3501 et seq.
